# Electrokinetic Motion of Neurotransmitter Ions through a 1.01 nm Diameter Single-Walled Carbon Nanotube

**DOI:** 10.1021/acs.jpcc.4c07482

**Published:** 2025-03-11

**Authors:** Mark D. Ellison, Jacqueline Allen, Michael Bonfiglio, Matthew Seeburger, Jean Setenet, Biagio DiGinto, Harrison Bonanny, Aaliyah Russell, David Baird, Liana Davis, Ella McCarthy, Alyson Manley, Sarah Blatt, David Lippe, Daniel Ragone, Brock Dyer, Jillian Osgood, Michael S. Strano

**Affiliations:** † Department of Chemistry, Ursinus College, 601 E. Main St., Collegeville, Pennsylvania 19426, United States; ‡ Department of Chemical Engineering, Massachusetts Institute of Technology, 77 Massachusetts Ave., Cambridge, Massachusetts 02139, United States

## Abstract

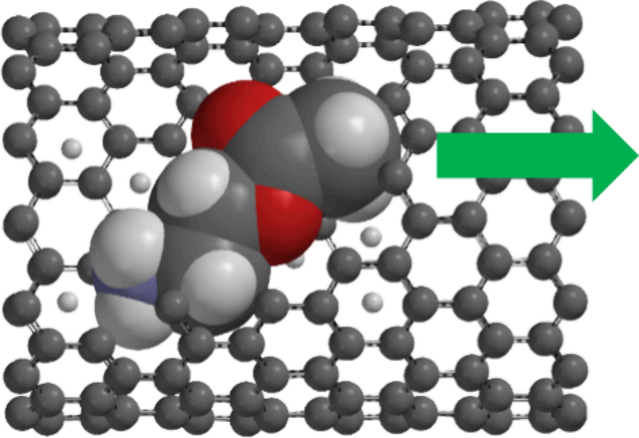

The transport of cations of the neurotransmitters acetylcholine, choline, and dopamine through a 1.01 nm-diameter, 1.1 mm-long single-walled carbon nanotube (SWNT) has been studied for the first time. As a comparison, sodium and aniline ion transport was also investigated. All of these ions exhibited significantly enhanced electrophoretic mobilities over bulk transport. The electrophoretic mobilities of acetylcholine, choline, and sodium were found to depend on pH, specifically increasing as pH decreases. This result is explained by hydrogen ions saturating the surface charges of the SWNT. Conversely, dopamine and aniline have mobilities that do not depend on pH. This difference is attributed to the benzene ring and the size of these ions. An analysis of the time required for an ion to traverse the nanotube shows that the ions adsorb to and desorb from the walls as they pass through the tube. Acetylcholine, choline, and sodium show desorption rate constants that decrease with increasing pH, whereas dopamine and aniline have rate constants that remain constant over different pH values. This is consistent with the relationship between adsorption and desorption rate constants and mobility from an adsorption/desorption kinetic model.

## Introduction

The motion of ions and molecules through openings with nanometer-sized dimensions (nanopores) is important for understanding transport in confined spaces and has applications in separations, filtrations, and battery design. Of particular importance is the motion of molecules and ions through sub-10 nm nanopores, referred to as single-digit nanopores (SDNs).^1^ This field of study has seen significant advances recently, but gaps in our knowledge remain.^2^

Nanopores have been constructed of a variety of different materials, including graphene,^3−6^ SiC,^7−10^ Si/SiN,^11−19^ MoS_2_,^20^ and carbon nanotubes.^21−31^ Carbon nanotubes offer a number of significant nanopore characteristics, including a high aspect ratio, hydrophobic interior, unexpectedly rapid transport of water,^32−35^ and selectivity of ion transport.^23,36−38^ The motion of alkali metal cations through single-walled carbon nanotubes (SWNTs) has been well-studied,^21−24,39^ and passage of cations through an SWNT is characterized by a two-state Coulter effect in the current through the SWNT. These ions exhibit electrophoretic mobilities that are significantly greater in SWNTs than in bulk water.^23,39^ Their motion is also highly dependent on nanotube diameter.^22^ Recently, we studied the transport of the neutral molecule methanol^39^ and several amino acid cations^40^ through a 2.25 nm-diameter SWNT. The methanol was found to be pushed through the SWNT by collisions with H^+^ ions that were electrically driven through the nanotube. The amino acid cations were themselves driven through the nanotube and also exhibited adsorption/desorption behavior on the nanotube sidewalls. Clearly, the motions of species larger than monatomic ions have interesting and rich dynamics.

One knowledge gap that exists is the motion of medium-sized molecule-ions through SWNTs. As noted previously, the motion of monatomic ions has been studied, as has the motion of large biomolecules such as proteins and DNA.^25,41,42^ The study of biomolecule transport through SDNs has focused primarily on detection and sequencing,^4−6,12,14,16,25,43−50^ leaving the details of motion largely unexplored. Therefore, to better understand the transport of medium-sized molecular cations through an SWNT SDN, we studied the motions of choline, acetylcholine, dopamine, and aniline hydrochlorides through a 1.0 nm-diameter SWNT.

A search of the literature reveals that nanopores have been developed to detect low concentrations of dopamine^51−54^ or acetylcholine.^55^ Therefore, this study is, to our knowledge, the first to investigate the dynamics of molecular motion of neurotransmitter cations through an SWNT SDN. We note that fundamental understanding of the molecular motion through an SWNT could lead to applications such as the ability to precisely (both quantitatively and spatially) deliver these neurotransmitters with an SWNT.

## Experimental Section

The construction of the SWNT nanopore devices has been described previously.^22,23,39,40^ Briefly, a catalyst solution was painted on the edge of a rectangular piece of Si wafer. Chemical vapor deposition (CVD) was used to grow SWNTs using CH_4_ as the source of carbon. This process yields SWNTs that are millimeters to centimeters in length, roughly aligned, and spaced about 50–250 μm apart. Then, the SWNTs were characterized using scanning electron microscopy (SEM) and Raman spectroscopy. A 3 mm-thick sheet of polydimethylsiloxane (PDMS, Dow Sylgard, 10:1 elastomer to curing agent ratio) mask was created. Holes of dimension 1 × 1 mm were manually punched in the PDMS sheet, spaced 1–1.5 mm apart. Then, the sheet was glued with PDMS glue (Dow Sylgard, 3:1 elastomer to curing agent ratio) to the wafer. After the glue had cured, 4.0 M nitric acid was placed in the holes in the PDMS sheet for 30 min to etch away the exposed SWNTs. This also produces carboxylic acid functional groups on the ends of the SWNTs.^56^ The reservoirs were then thoroughly rinsed with ultrapure water (Millipore, 18.2 MΩ cm).

**Figure 1:**
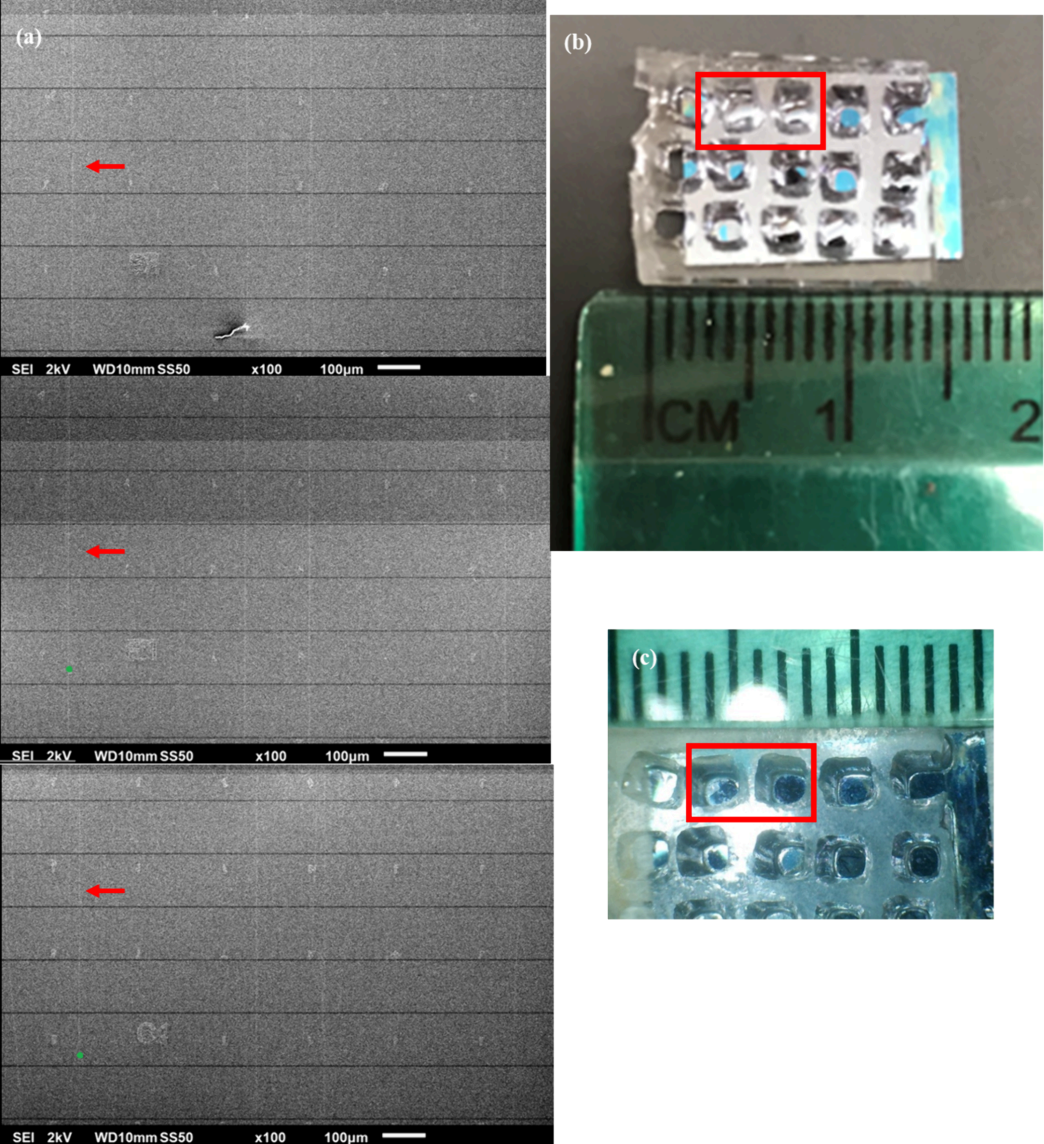
(a) SEM image of the SWNT on a Si chip used in this research. Red arrows indicate the SWNT. (b) Visual image (approx 2×) of the Si chip with a PDMS cover attached. (c) Visual image (approximately 10×) of the Si chip with the PDMS cover. Red rectangles indicate the reservoirs used in the relevant experiments.

SEM and light microscopy images of the device are shown in Figure 1. Panel a shows three SEM images of the sections of the Si chip where the nanotube was located. The SWNT appears as a faint white vertical line, so it has been indicated with red arrows. (A full image of the entire Si chip is shown in the Supporting Information Figure S1.) Panel b shows an optical image of the device (approximately 2× magnification), with the red rectangle indicating the adjacent reservoirs used in this research. This pair was measured to be 1.1 ± 0.1 mm apart, and the SWNT was assumed to be equal to this length. Panel c shows an optical image of the device at approximately 10× magnification. A Raman spectrum of the SWNT before attachment of the PDMS (Figure S2) found a radial breathing mode peak at 246.22 cm^–1^, which corresponds to a diameter of 1.01 nm.^57^

The device was placed on optical mounting hardware on a nitrogen-cushioned vibration isolation table surrounded by a Faraday cage (Kinetic Systems). This setup reduces the electronic noise to 2 pA or lower. Ag/AgCl electrodes were produced by immersing Ag wire in bleach for 20 min. The applied voltage was controlled by a Molecular Devices Axopatch 200B amplifier, and the current was collected using a Molecular Devices Digidata 1550A D/A converter using Clampex software set to 2 kHz Bessel low-pass filter and 250 kHz acquisition frequency. Data were filtered in ClampFit software with a boxcar low-pass filter using 99 smoothing points. Pore-blocking current (PBC) and dwell times were obtained using ClampFit software, and statistical analysis of these data was performed in Igor (Wavemetrics). At least 50 pore-blocking events were collected and analyzed at each voltage or pH value.

**Chart 1:**
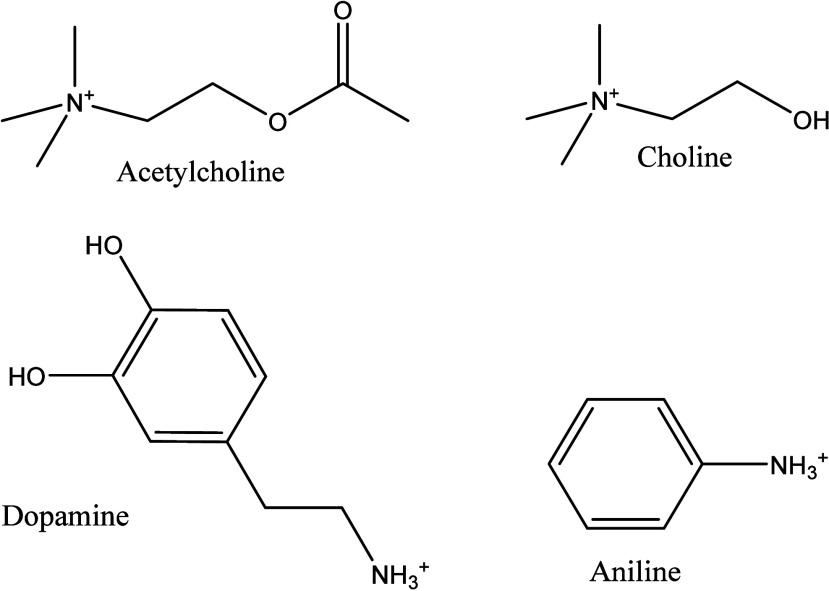
Chemical Structures of the Cations of Acetylcholine, Choline, Dopamine, and Aniline

Solutions were made by dissolving choline hydrochloride (Aldrich, ≥99%), acetylcholine hydrochloride (Aldrich, ≥99%), dopamine hydrochloride (Aldrich, ≥99%), aniline hydrochloride (Aldrich, ≥99%), potassium chloride (Aldrich, 99.999%), or sodium chloride (Aldrich, 99.999%) in ultrapure water (Millipore, 18.2 MΩ cm) to make 1.0 M solutions. Chemical structures of the molecular ions are shown in Chart 1. The pH of each solution was adjusted by adding 3.0 M hydrochloric acid in 2-μL increments until the desired pH was reached. The pH was measured with a Fisher Scientific AB15 pH meter.

## Results and Discussion

**Figure 2:**
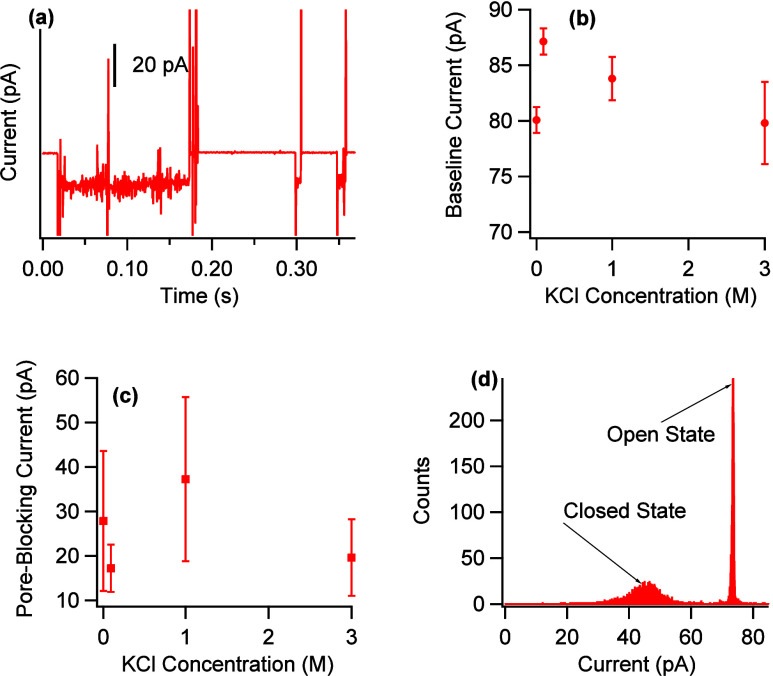
(a) Current data for 0.10 M KCl at 1000 mV showing pore-blocking events. (b) Baseline current at 1000 mV for different KCl concentrations. (c) Pore-blocking current at 1000 mV for different KCl concentrations. (d) Histogram of current data from panel a showing an open state (right peak) and a closed state (peak to the left of the open state).

Because the SWNT is covered with a piece of PDMS, it is important to establish that the observed transport is through the SWNT and not through nanometer-sized fissures in the PDMS. Previous work has established that motion through PDMS is clearly distinguishable from that through an SWNT.^58^ Specifically, for transport through PDMS, the baseline current and pore-blocking current (PBC) depend strongly on the solute ion concentration, whereas for an SWNT there is no dependence. Additionally, for PDMS, a histogram of current values will show multiple Coulter states, whereas for SWNTs, it will show just two distinct states corresponding to an open or closed pore. Figure 2 shows these data for the device used in this research. Panel a shows the current data when KCl solutions were placed in adjacent reservoirs and a voltage of 1000 mV was applied. Panel b shows the baseline current as a function of potassium ion concentration. Potassium was used because its transport through SWNTs and PDMS is well-understood. The baseline current does not depend on the potassium ion concentration, which is consistent with motion through an SWNT and inconsistent with motion through PDMS. Panel c shows the PBC as a function of potassium ion concentration. Once again, there is no dependence, which is consistent with transport through an SWNT and inconsistent with transport through PDMS. Finally, panel d shows a histogram of the current values for the trace in panel a. The histogram clearly shows two distinct states, corresponding to an open nanotube and one that is blocked by the passage of an ion. This is consistent with transport through an SWNT; transport through PDMS shows more than two states. Therefore, these data demonstrate that the transport studied in this research is that of motion through an SWNT.

Control experiments (Figure S3) of just ultrapure water in the reservoirs, no water in the reservoirs, and wells not connected by SWNTs show level baselines with no pore-blocking or other events. These experiments establish that the two-state Coulter events observed with neurotransmitter solutions are due to the neurotransmitter ions.

**Figure 3:**
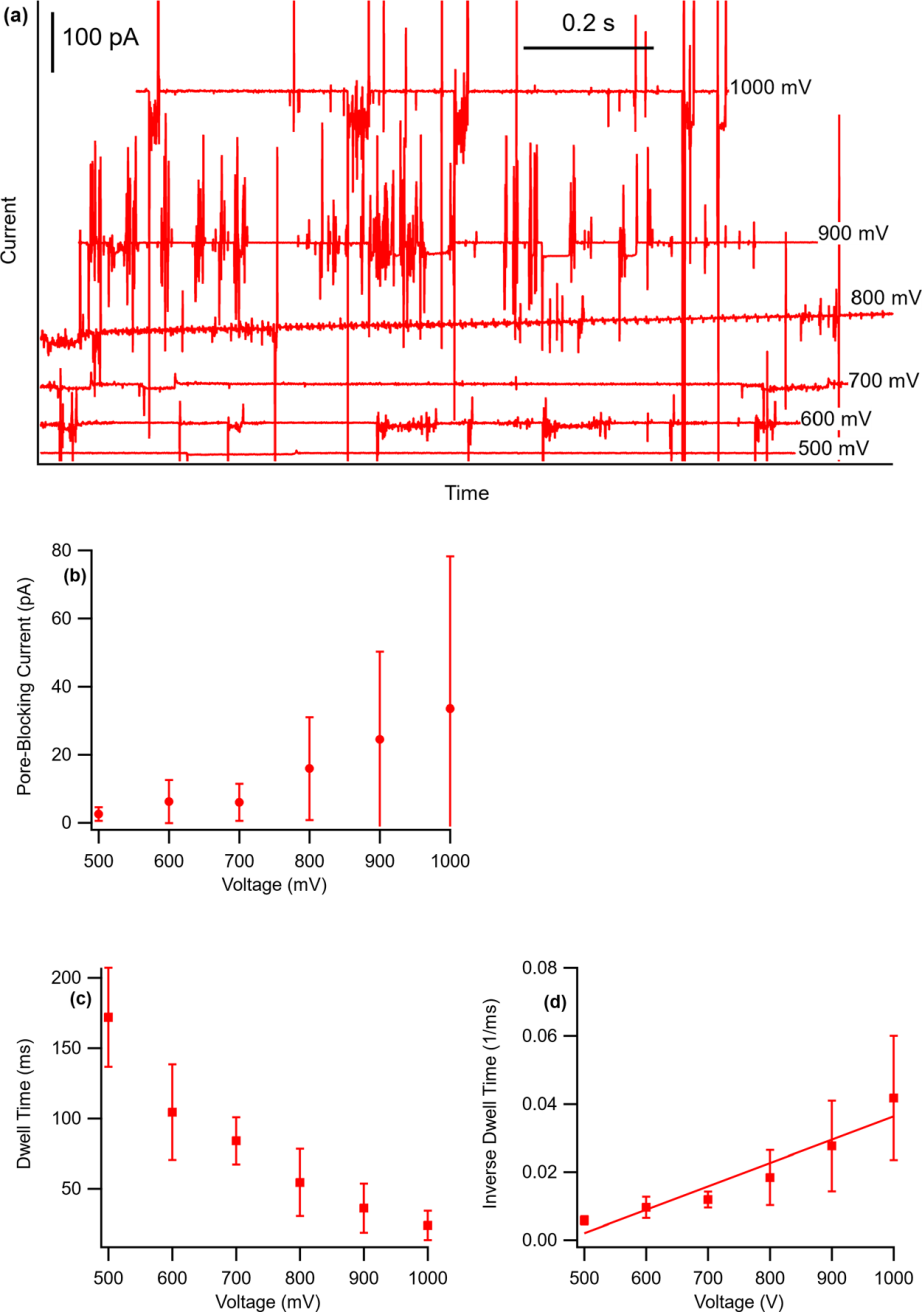
(a) Sections of raw current traces for acetylcholine at pH 3.45 showing events and noise. The different voltages are indicated. (b) Pore-blocking current of acetylcholine as a function of voltage. (c) Dwell time of acetylcholine as a function of voltage. (d) Inverse dwell time as a function of voltage. The mobility of acetylcholine is proportional to the slope.

Current traces for acetylcholine at pH 3.45 are shown in Figure 3a. No pore blocking was observed below 500 mV, which is called the threshold voltage for this molecule. As noted previously, the nitric acid treatment adds carboxylic acid groups to the pore mouths. The electronegative oxygen atoms cause this region to have a negative electrical charge, which attracts cations and rejects anions. The minimum voltage needed to overcome this attraction and push the ions through the SWNT is known as the threshold voltage. Figure 3b shows that the PBC (the decrease in current during an event) increases with increasing voltage, in agreement with previous studies.^22,24,39,40^Figure 3c shows that there is an inverse relationship between the dwell time and the voltage, which is characteristic of an ion that has a constant electrophoretic mobility. The graph of the inverse dwell time versus voltage, shown in Figure 3d, is a straight line whose slope is proportional to that ion’s mobility:  or , where *L* is the length of the SWNT, *V* is the applied voltage, and *t* is the dwell time. Mobilities for the ions, except for dopamine, were determined from the slope of graphs similar to Figure 3d. Dopamine exhibited pore blocking only at 1000 mV, so its mobility was determined by directly solving the equation . Finally, the data in Figure 3b–d are all indicative of the motion of ions through an SWNT, confirming the earlier conclusion.

PBCs and dwell times as a function of voltage for choline and sodium are shown in Figures S5 and S6. These cations display the same behavior as acetylcholine: PBC increases with increasing voltage, and dwell time decreases with increasing voltage. From the graphs of inverse dwell time versus voltage, mobilities were determined. Dopamine and aniline were found to have a threshold voltage of 1000 mV, so it is not possible to make graphs like Figure 3 for those ions.

**Figure 4:**
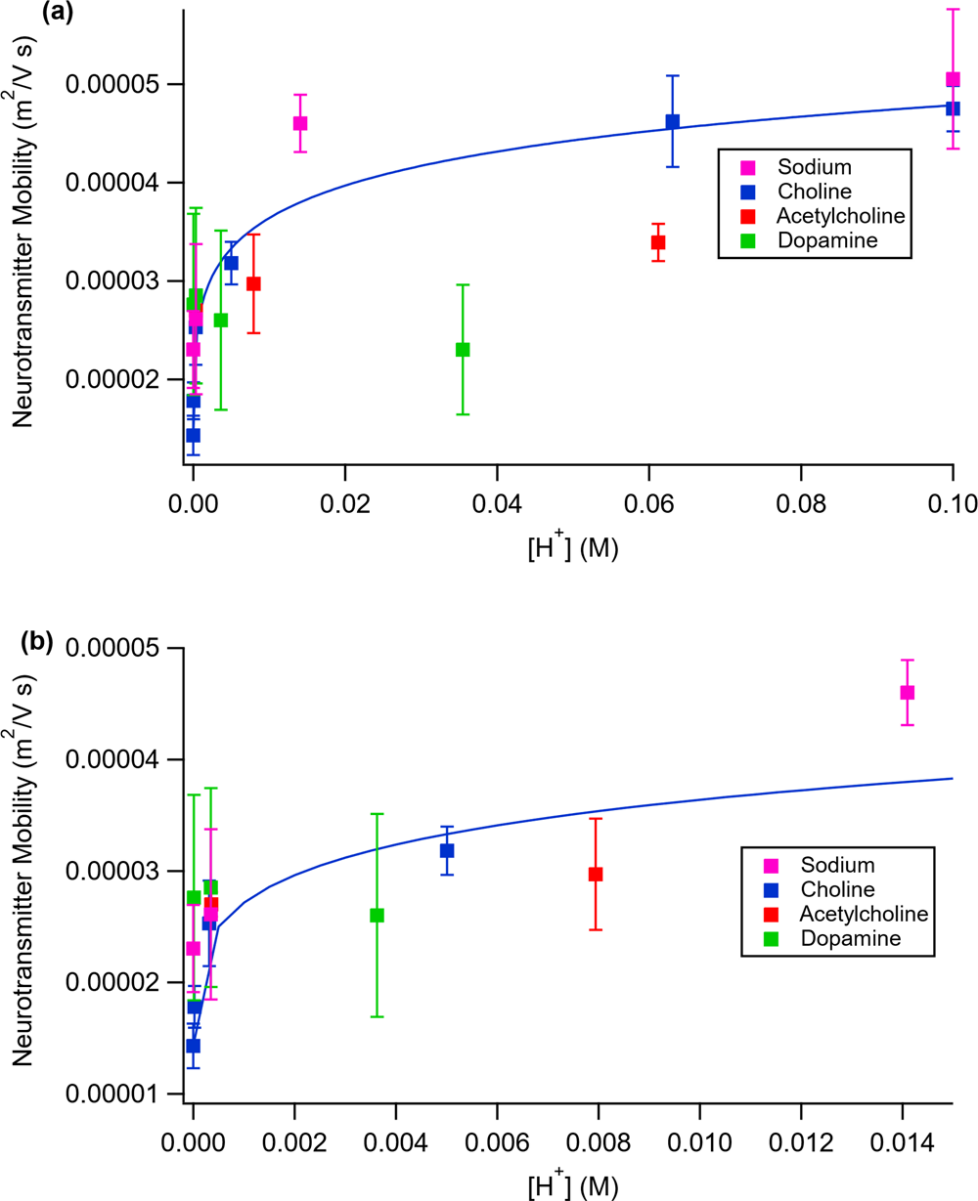
(a) Ionic mobilities of sodium choline, acetylcholine, and dopamine cations through a 1.01 nm SWNT as a function of solution pH. The line is a fit to guide the eye. (b) Close-up of the low-hydrogen-concentration (high pH) region of panel a.

It was noticed early in the research that the mobilities of the neurotransmitter ions correlated with the inherent pH of the 1.0 M solutions of these ions (Figure S4). Therefore, to determine whether this observation was a real effect, the mobilities of the ions were measured at different pH values. The maximum pH is limited by the pH of a solution at a concentration of 1.0 M, but the pH can be lowered by adding hydrochloric acid. However, whereas choline was stable at a pH of 1.0, acetylcholine and dopamine would undergo decomposition at that pH, so they could not be tested at that level of acidity. Figure 4 shows the mobilities of acetylcholine, choline, and dopamine as a function of hydrogen ion concentration. For acetylcholine and choline, the mobility increases as [H^+^] increases (pH decreases) and levels off at [H^+^] ≈ 0.05–0.06 M. To test whether these results extended to previously studied ions, 1.0 M NaCl solutions were tested. As shown in Figure 4, Na^+^ mobility also increases as [H^+^] increases and levels off at approximately the same value of [H^+^]. This indicates that the trend is shared by both complicated and simple ions. On the other hand, dopamine did not exhibit a mobility that increased and leveled off. Rather, its mobility was essentially constant over the [H^+^] range studied.

**Figure 5:**
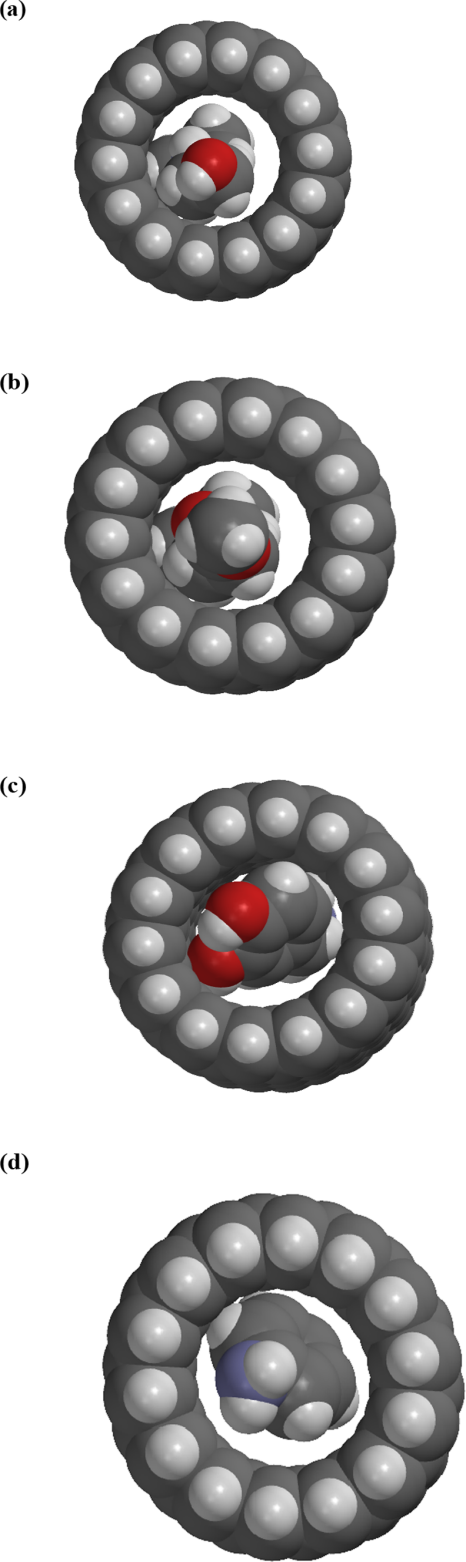
Space-filling models of (a) choline, (b), acetylcholine, (c) dopamine, and (d) aniline inside an SWNT. The gray spheres are carbon atoms, white spheres are hydrogen, blue spheres are nitrogen, and red spheres are oxygen.

At any pH, the general pattern for mobility is sodium > choline > acetylcholine > dopamine. This trend inversely correlates with molecular size. Sodium ions are much smaller than the neurotransmitter ions and therefore have a higher mobility. Figure 5 shows space-filling models of choline, acetylcholine, dopamine, and aniline inside a segment of a 1.0 nm-diameter SWNT. Qualitatively, as the molecular size of these ions increases, larger ions are observed to have lower mobilities through SWNTs, which agrees with previous studies.^23^

The mobilities of the various ions in this study are presented in Table 1 and compared to those in other SWNT systems and in bulk water. The most notable observation is that the mobilities through SWNTs are much higher than those in bulk water. This result has a 2-fold explanation. First, motion through an SWNT is essentially confined to the direction of the electric field along the tube axis; motion in directions perpendicular to the electric field is largely constrained. Conversely, in bulk water the ion generally moves along the direction of the electric field but sideways motion is still possible, which decreases the observed bulk mobility. Second, the minimal friction between the moving ion and the SWNT wall enhances transport.^38,59^

**Table 1:** Mobilities of Ions in This Study and Comparisons to Previous Studies

ion	mobility (m^2^/V s)	pH	SWNT diameter (nm)
Na^+^ (ref (23))	5.0 × 10^–8^	7	1.5
K^+^ (ref (23))	5.0 × 10^–8^	7	1.5
Na^+^ (this work)	2.3 × 10^–5^ ± 3.9 × 10^–6^	7	1.01
choline (this work)	1.4 × 10^–5^ ± 2.0 × 10^–6^	7	1.01
choline (ref (60))	3.9 × 10^–8^	7	bulk
acetylcholine (this work)	2.7 × 10^–5^ ± 1.1 × 10^–6^	3.45	1.01
acetylcholine (ref (60))	3.9 × 10^–8^	7	bulk
dopamine (this work)	2.76 × 10^–5^ ± 9.2 × 10^–6^	4.85	1.01
dopamine (ref (61))	3.17 × 10^–8^	7.0	200 μm-wide microchannel
aniline (this work)	2.27 × 10^–5^ ± 6.7 × 10^–6^	2.48	1.01

Figure 4 shows that acetylcholine, choline, and sodium have mobilities that depend on the pH of the solution, whereas dopamine does not. We will first address the observation of pH-dependent mobilities and then discuss the different behavior of dopamine.

Several mechanisms could explain the pH dependence of the mobilities of sodium, choline, and acetylcholine. First, the hydrogen ions could saturate the carboxylic acid groups at the entrance of the pore mouth, making it easier for the cations to enter the SWNT. Because the pore entrance of a 1.01 nm-diameter SWNT can have at most 14 carboxylic acid groups, it would not take a large number of H^+^ ions to saturate them. Also, the p*K*_a_ of these carboxylic acids is about 4.5,^62^ so they should be essentially fully saturated at a pH of 3.5, which is 2 orders of magnitude higher than the observed saturation pH of about 1.4–1.7. Furthermore, saturation of the carboxylic acid groups at the pore mouth should decrease the threshold voltage, but no such decrease was observed. Therefore, the saturation of pore-mouth carboxylic acid groups could be part of the observed effect but cannot be the full explanation.

Second, collisions from H^+^ ions could increase the velocity of an ion in an SWNT. Assuming a complete momentum transfer, a H^+^ ion colliding with a sodium, choline, or acetylcholine ion would increase the latter’s velocity by 21%, 10%, or 7.1%, respectively. The collision cross section of these ions in an SWNT is about the size of 4–6 H^+^ ions. Therefore, the maximum velocity increase for these ions due to collision from H^+^ ions is 30–100%. The observed increase in mobilities is 250%, so direct momentum transfer cannot explain the full effect. Furthermore, molecular dynamics simulations show the importance of collisions with water molecules during ion transport through SWNTs.^63−65^ Any momentary increase in velocity from a H^+^ ion is likely to be quickly transferred to a water molecule ahead of the ion, and the increase in velocity would not be sustained. Therefore, collisional momentum transfer is not a likely explanation for the observed mobility dependence on pH.

Third, the role of surface charge in ion transport through nanopores is known to be quite important.^66−72^ Charges at the pore wall directly influence the internal motion of ions, as does adsorption of counterions.^66,67,69,73^ The observed increase and plateau in ion mobility with increased hydrogen ion concentration suggests that H^+^ ions are adsorbing on the SWNT wall. The SWNT in the device is sandwiched between SiO_2_ and PDMS. Both of these materials have negatively charged oxygen atoms that can induce a negative charge in the SWNT wall, which would create a driving force for H^+^ adsorption. With little H^+^ adsorption at higher pH, the neurotransmitter ions would be attracted to the surface charge of the SWNT and exhibit a lower mobility. On the other hand, at lower pH, the surface charge would be passivated by adsorbed H^+^ ions, allowing the neurotransmitter ions to pass with a greater velocity and therefore a larger mobility.^63,69,71,73−78^

At the saturation H^+^ concentration of about 0.050 M, assuming that the concentration of H^+^ in the SWNT is the same as in the bulk, the number of hydrogen atoms inside the SWNT is 2.6 × 10^4^. Dividing by the surface area of the 1.01 nm diameter, 1.1 mm long SWNT gives 0.0012 C/m^2^, which is in excellent agreement with the surface charge of 0.0015 C/m^2^ determined for SWNT nanopore devices of very similar construction.^22,23^ It also agrees well with the surface charge used in a molecular dynamics simulation that found strong cation adsorption on the surface charge.^73^ This is good evidence for the H^+^ adsorption mechanism.

**Figure 6:**
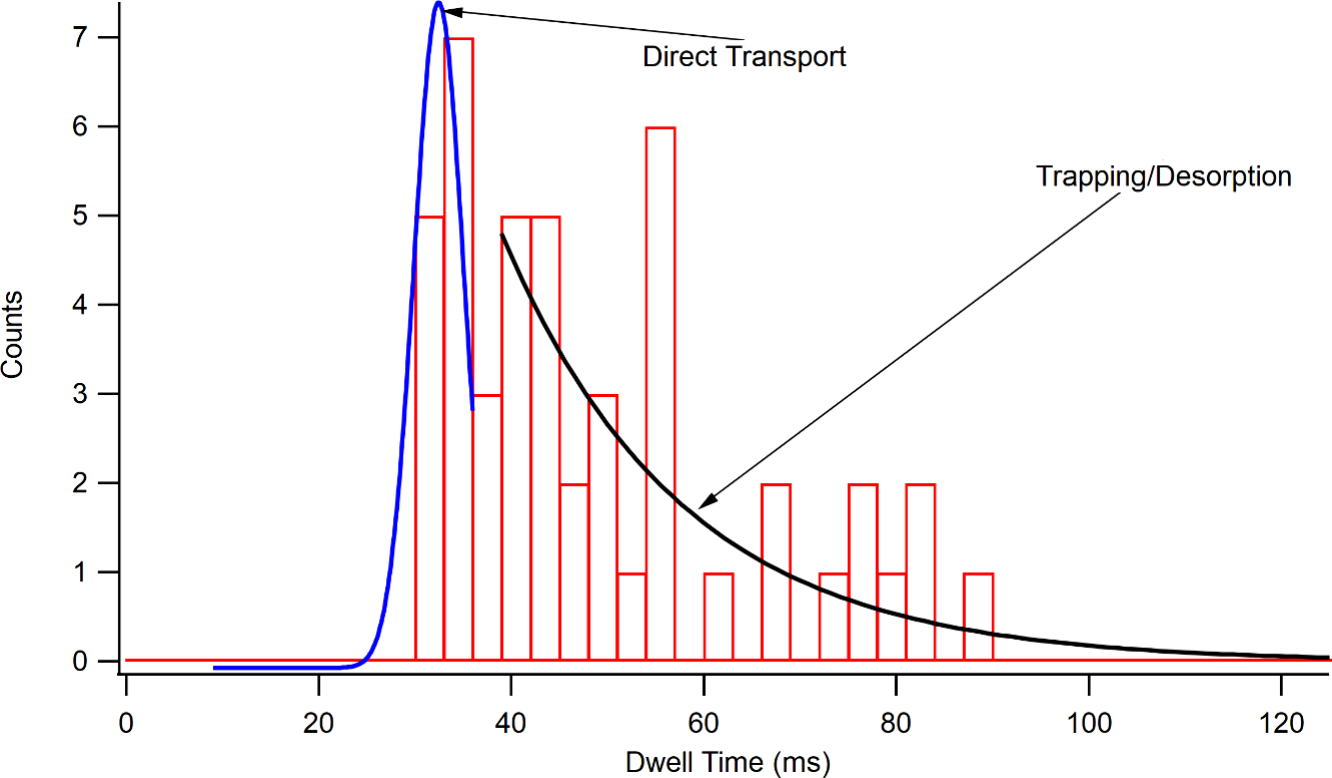
Dwell time distributions for dopamine at pH 4.85. The short dwell times correspond to direct transport, and the long dwell times correspond to ions that trapped and desorbed as they traveled through the SWNT. The blue and black lines are fits to guide the eye, and the black line’s fit is used with eq 2 to find *k*_des_.

More evidence for the H^+^ adsorption model comes from the dwell time data. As shown in Figure 6, the distribution of dwell times for dopamine shows an initial peak that corresponds to direct transport through the SWNT. There is also a tail of slowly decreasing counts of dwell times. All ions studied displayed a similar distribution of dwell times. We observed similar data in a previous study on the transport of amino acid cations through an SWNT.^40^ A model developed by Yeh and Hummer^79^ to describe the molecular dynamics simulation of RNA transport through SWNTs was used to describe those data, and we applied it again to this work. In brief, the average time for a molecule to pass through at SWNT has one contribution from the drift velocity due to the electric field and one from the adsorption/desorption process, as given in the equation
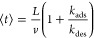
1where *L* is the length of the SWNT; *v* is the drift velocity; and *k*_ads_ and *k*_des_ are the rate constants for adsorption and desorption, respectively. The desorption rate constant is related to the time constant of the exponentially decreasing tail of the dwell times by
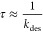
2

Full details of the analysis are provided in the Supporting Information. Table 2 reports several of the adsorption and desorption rate constants obtained from the data. It is noteworthy that the values of the rate constants for the neurotransmitters are similar to those previously reported for amino acids,^40^ confirming the applicability of the model. Several trends are apparent in the rate constants in Table 2. First, as pH decreases, *k*_ads_ also decreases. In contrast, *k*_des_ shows no statistically significant variation with pH. Finally, *k*_ads_ for dopamine is constant over the pH range under which it was studied.

**Table 2:** Adsorption and Desorption Rate Constants for the Cations in This Study

solute	pH	*k*_ads_ (s^–1^)	*k*_des_ (s^–1^)
Na^+^	7.0	41.3 ± 13.4	52.5 ± 17.0
Na^+^	3.46	21.4 ± 11.0	23.0 ± 11.8
Na^+^	1.85	10.3 ± 16.2	8.64 ± 13.6
choline	7.0	69.6 ± 20.0	121 ± 35
choline	3.5	40.2 ± 5.2	130 ± 17
choline	1.3	95.3 ± 14.3	169 ± 25.3
acetylcholine	3.45	253 ± 59	221 ± 52
acetylcholine	2.10	160 ± 80	17.9 ± 9.0
acetylcholine	1.21	57.0 ± 8.6	55.5 ± 8.4
dopamine	4.85	27.5 ± 4.6	53.6 ± 9.0
dopamine	3.46	22.7 ± 3.7	46.8 ± 7.6
dopamine	2.44	29.4 ± 6.3	46.2 ± 9.9
dopamine	1.45	22.7 ± 3.8	24.4 ± 4.1
aniline	2.48	19.2 ± 12.8	15.7 ± 10.5
aniline	1.70	22.7 ± 8.2	23.0 ± 8.3
aniline	1.07	17.1 ± 10.4	21.7 ± 13.2

The decrease in *k*_ads_ with decreasing pH indicates that the neurotransmitter adsorption process is slower when more H^+^ ions are present in the nanotube. This result is consistent with the proposed model of H^+^ adsorption. With more positively charged hydrogen ions near the SWNT wall, the adsorption of a positive neurotransmitter ion would have a higher activation energy as a result of Coulombic repulsion, which would result in a reduced value of the adsorption rate constant.

Qualitatively, *k*_ads_ increases with increasing molecular size, with the exception of dopamine. We attribute this exception to the size of dopamine relative to the internal channel of the SWNT. Panels a–c of Figure 5 show space-filling models of choline, acetylcholine, and dopamine, respectively, inside a 1.0 nm-diameter SWNT. Choline and acetylcholine occupy much of the space inside the nanotube but still have some room to rotate and tumble. On the other hand, dopamine takes up almost all of the internal space and has essentially no room for any motion except translation directly down the nanotube axis. Once inside the SWNT, the dopamine ion can only move to the other end. Even adsorption on the tube walls is difficult for dopamine. For the other neurotransmitters, as the hydrophobic portion of the molecule increases, so does *k*_ads_. A similar result was found in our studies with amino acids,^40^ which indicated that the hydrophobic part of the molecules is strongly involved in the adsorption to the SWNT wall.

A search of the literature revealed very few studies about the motion of polyatomic ions through an SWNT nanopore. Yeh and Hummer calculated *k*_ads_ and *k*_des_ for RNA hexamers (adenine, A_6_, and uracil, U_6_) that are 8 orders of magnitude higher than the ones found here. However, the electric fields used in their simulations were much higher than those used in our experiments (∼5 × 10^8^ V/m compared to ∼ 1 × 10^3^ V/m). Their RNA hexamers are also much larger than the neurotransmitters studied and have much larger surface areas for interaction with the SWNT wall. Otherwise, the literature search found no other results for comparison. Molecular dynamics simulations are very effective at illustrating the details of ionic and molecular motion through an SWNT,^63,65,80−83^ and we hope that the experimental results reported here will encourage molecular dynamics simulations of this kind of system.

We now turn to the observation that dopamine’s mobility is not pH-dependent. An obvious factor is its molecular structure. Sodium is a simple monatomic cation, and acetylcholine and choline are alkyl-chain cations. Dopamine, on the other hand, has a benzene ring as part of its structure, making it rather different from the other ions. In order to test this difference, we also tested aniline hydrochloride, which, like dopamine, has a benzene ring. Although aniline has some similarities to dopamine, it lacks the hydroxyl groups and alkyl chain of dopamine. Therefore, a comparison of the two will elucidate the importance of the benzene ring in the motion of these ions.

**Figure 7:**
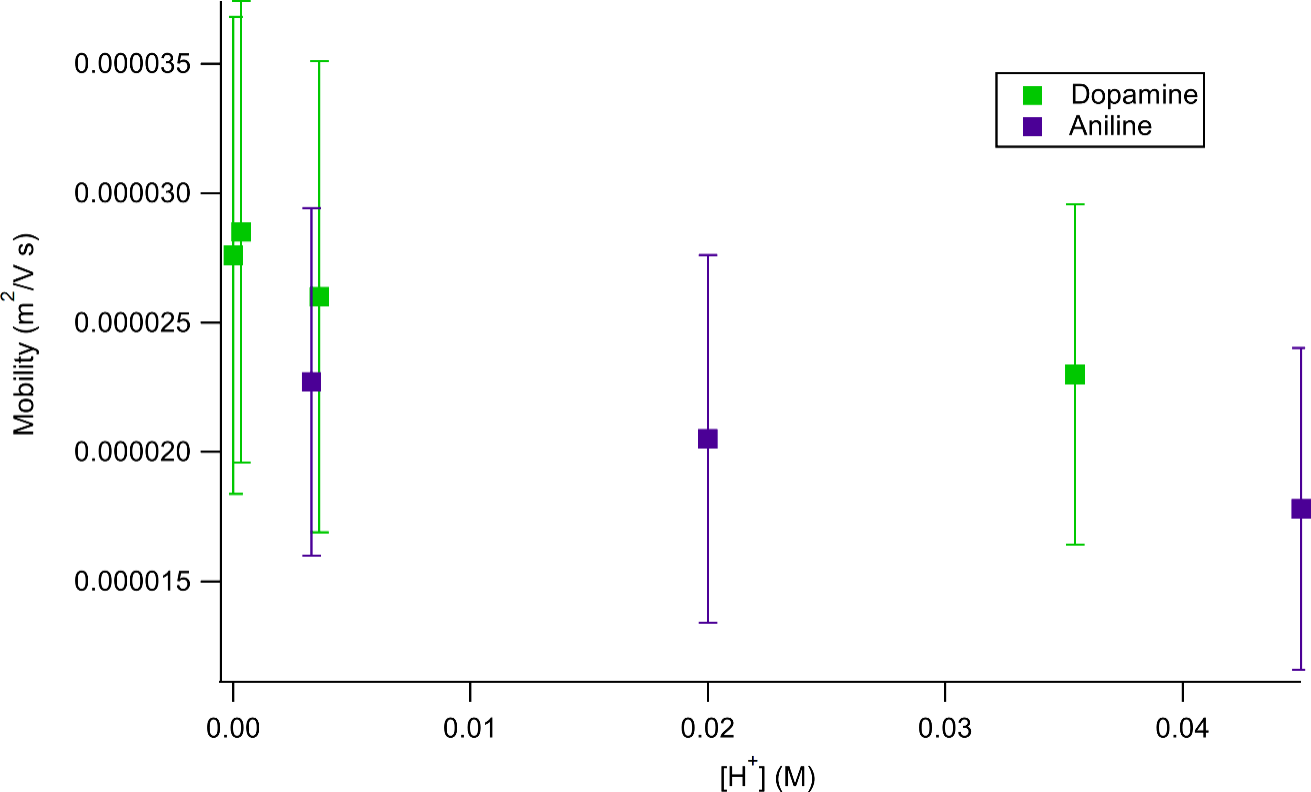
Ionic mobilities of dopamine (green) and aniline (purple) cations through a 1.01 nm-diameter SWNT as a function of pH.

Figure 7 compares the mobilities of the aniline and dopamine cations at different pH values. The two ions show no statistically significant changes as a function of pH. This result suggests that the structural similarity of these molecular ions, namely, the benzene ring, plays a role in their transport behavior. Additionally, although there is overlap of the error bars, the average values of the mobilities for aniline are slightly lower than those of dopamine, indicating the possibility of slower motion through the SWNT.

The kinetic data reported in Table 2 shed further light on the motion of these ions. As was the case for dopamine, the *k*_ads_ values for aniline are essentially the same at different pH values. Also, the values of *k*_ads_ for dopamine and aniline cations are, within the uncertainties, the same. The closeness of *k*_ads_ for dopamine and aniline cations suggests that the same structural feature in the molecules, namely, the benzene ring, plays a key role in the adsorption of these ions to the SWNT wall. Pi–pi interactions can lead to attractions that are strong enough to noncovalently functionalize carbon nanotubes,^84^ so it is possible that those interactions allow dopamine and aniline to adsorb to the inner wall of the SWNT. Examination of Figure 5c,d shows that the space occupied by the dopamine or aniline molecule puts it in an almost-adsorbed configuration, possibly facilitating adsorption. Additionally, because the dopamine and aniline molecules each occupy almost all of the internal space of the SWNT, it is possible that their motion would dislodge any adsorbed hydrogen ions, negating their screening effect.

Several other points from the aniline data can be noted. First, *k*_des_ values for aniline are significantly lower than those for dopamine and the rest of the ions. This result means that once aniline ions are adsorbed, it takes them much longer to desorb, and, consequently, to move through the nanotube. This could account for the lower mobility of aniline compared to dopamine and the other ions. Second, if the expression for velocity and mobility is inserted into eq 1 and then rearranged, the following equation is obtained:
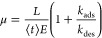
3

*L* is constant, and for a given electric field, *E*, there are several possibilities for a constant mobility. First, if either *k*_ads_ ≫ *k*_des_ or *k*_ads_ ≪ *k*_des_, then the mobility will be essentially constant. That is not the case for this system. Second, if *k*_ads_ and *k*_des_ are constant with pH, then their ratio will be constant, and the mobility can be constant. This is the case for dopamine and aniline. Constant values of *k*_ads_ and *k*_des_ suggest that the activation energies of adsorption and desorption do not depend on pH. That is consistent with a different force, such as pi–pi interactions, being the primary driving force for adsorption and desorption.

## Conclusions

The study of the motion of neurotransmitter ions shows rich behavior. Acetylcholine, choline, dopamine, sodium, and aniline ions show enhanced electrophoretic mobility over bulk transport. Acetylcholine, choline, and sodium exhibit increasing mobilities as pH decreases, which is due to hydrogen ion adsorption on the sidewall of the SWNT that passivates surface charges. Dopamine and aniline have mobilities that do not change with changing pH, which is likely due to the benzene ring in their molecular structures. The analysis of the dwell times shows that the ions adsorb to and desorb from the SWNT wall as they move through the tube. The rate constants for adsorption for acetylcholine, choline, and sodium decrease with increasing pH, indicating a higher activation energy for adsorption when more hydrogen ions are present. The rate constants for dopamine and aniline are constant, indicating no change in the activation energy of adsorption.

## Supporting Information Available

SEM image; description of device construction; Raman spectrum; current traces for control experiments; mobilities of acetylcholine, choline, and dopamine; pore-blocking currents and dwell times of choline and sodium; dwell time distributions of sodium, choline, and acetylcholine; and calculation of adsorption and desorption rate constants, Neurotransmitter Paper (PDF)

## Abbreviations

SWNT: single-walled carbon nanotube
SDN: single-digit nanopore
PDMS: polydimethylsiloxane
PBC: pore-blocking current
