# Supplementary material for: Electrokinetic Motion of Neurotransmitter Ions through a 1.01 nm Diameter Single-Walled Carbon Nanotube
Source: J Phys Chem C Nanomater Interfaces. 2025 Mar 11;129(11):5472–82. doi: 10.1021/acs.jpcc.4c07482 (PMC11931543; doi:10.1021/acs.jpcc.4c07482)
Supplement: Supplementary file 1 — jp4c07482_si_001.pdf [file jp4c07482_si_001.pdf]

# Supporting Information for Electrokinetic Motion of Neurotransmitter Ions through a 1.01-nm Diameter Single-Walled Carbon Nanotube

*Mark D. Ellison*[1], Jacqueline Allen[1], Michael Bonfiglio[1], Matthew Seeburger,[1], Jean Setenet[1], Biagio DiGinto[1], Harrison Bonanny[1], Aaliyah Russell[1], David Baird[1], Liana Davis[1], Ella McCarthy[1], Alyson Manley[1], Sarah Blatt[1], David Lippe[1], Daniel Ragone[1], Brock Dyer[1], Jillian Osgood[1], and Michael S. Strano[2].*

[1]Department of Chemistry, Ursinus College, 601 E. Main St., Collegeville, PA 19426, USA.

[2]Department of Chemical Engineering, Massachusetts Institute of Technology, 77 Massachusetts Ave., Cambridge, MA 02139, USA.

**Table of Contents**


| **Content** | **Page** |
|---|---|
| Details of device construction | S1-S2 |
| Figure S1. SEM image | S3 |
| Figure S2. Raman spectrum of radial breathing mode | S4 |
| Figure S3. Control experiments: Current traces for ultrapure water, no water, and 1.0M KCl solutions in reservoirs not connected by a SWNT | S5-S6 |
| Figure S4. Mobilities of acetylcholine, choline, and dopamine | S7 |
| Figure S5. Pore-blocking current and dwell times of choline | S8 |
| Figure S6. Pore-blocking current and dwell times of sodium | S9 |
| Figure S7. Dwell Time Distributions of sodium, choline, acetylcholine, and aniline | S10-S11 |
| Calculation of $k_{ads}$ and $k_{des}$ | S12 |

| | |
|---|---|
| References | S12 |

**Details of device construction**

Regularly spaced markers were deposited on a silicon wafer using E-beam evaporation. Aligned SWNTs were then grown on sections of the wafer using methane CVD in a horizontal quartz tube furnace. (Thermo Fisher Scientific Lindberg/Blue M, TF55035A; Mass Flow controller: Aalborg SDPROC) Scanning electron microscope (SEM) images were collected to locate SWNTs on the wafer, as shown in Figure S1 below. A 3-mm-thick sheet of polydimethylsiloxane (PDMS, Dow Sylgard, 10:1 elastomer to curing agent ratio) mask was created. Holes of dimension 1mm×1mm were manually punched in the PDMS sheet, spaced 1–1.5 mm apart. Then, the sheet was glued with PDMS glue (Dow Sylgard, 3:1 elastomer to curing agent ratio) to the wafer. After the glue had cured, 4.0 M nitric acid was placed in the holes in the PDMS sheet for 30 min to etch away the exposed SWNTs. This also produces carboxylic acid functional groups on the end of the SWNTs.[1] The reservoirs were then thoroughly rinsed with ultrapure water (Millipore, 18.2 MΩ cm).

A Raman spectrum of the SWNT (Horiba LabRAM HR, 532 nm) collected before the PDMS was attached is shown in Figure S2 below and found a radial breathing mode peak at 246.22 $cm^{-1}$, which corresponds to a diameter of 1.01 nm.[2]

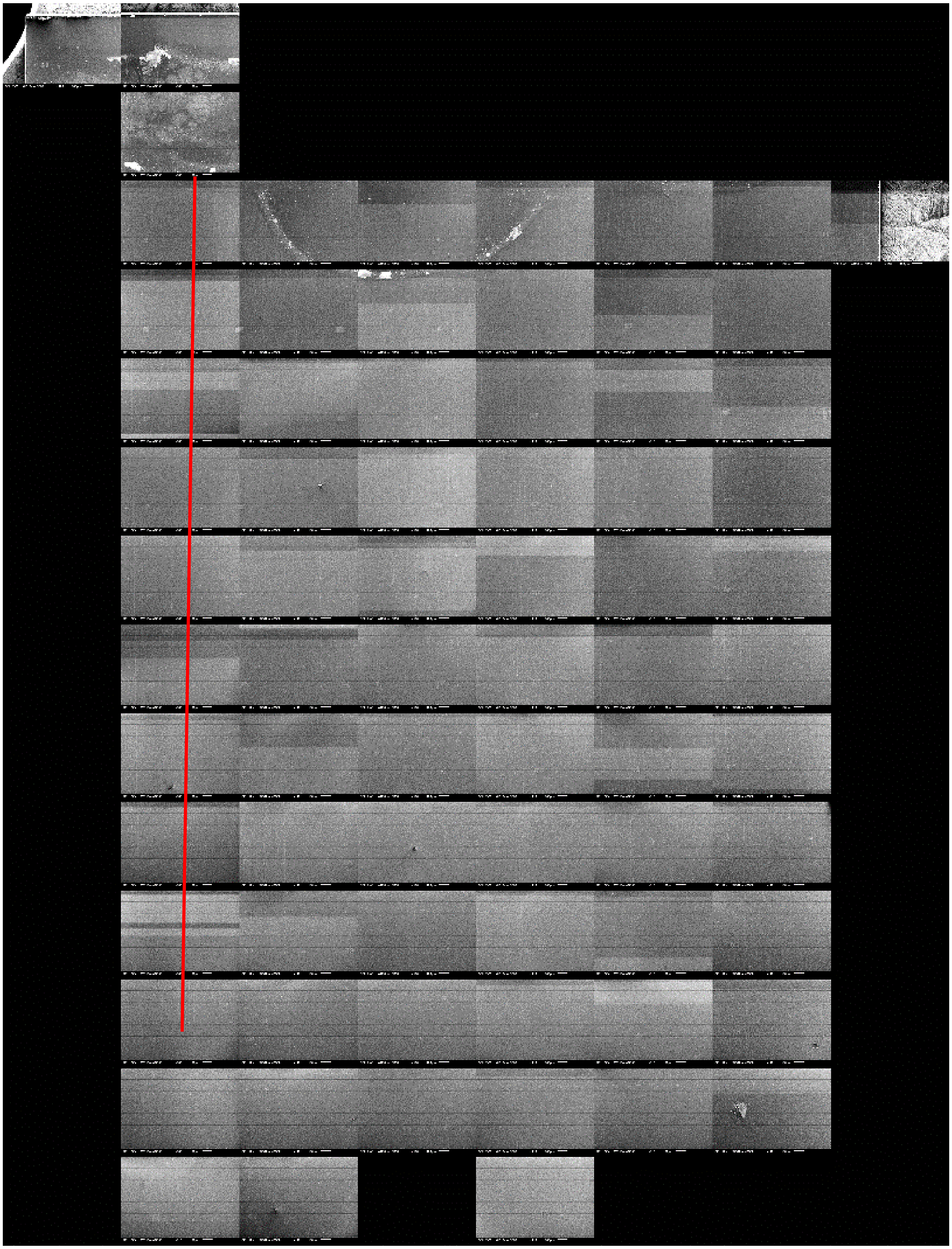

**Figure S1. Scanning electron microscope image of the silicon chip on which the SWNTs were grown. The SWNT in this study is highlighted by the vertical red line.**

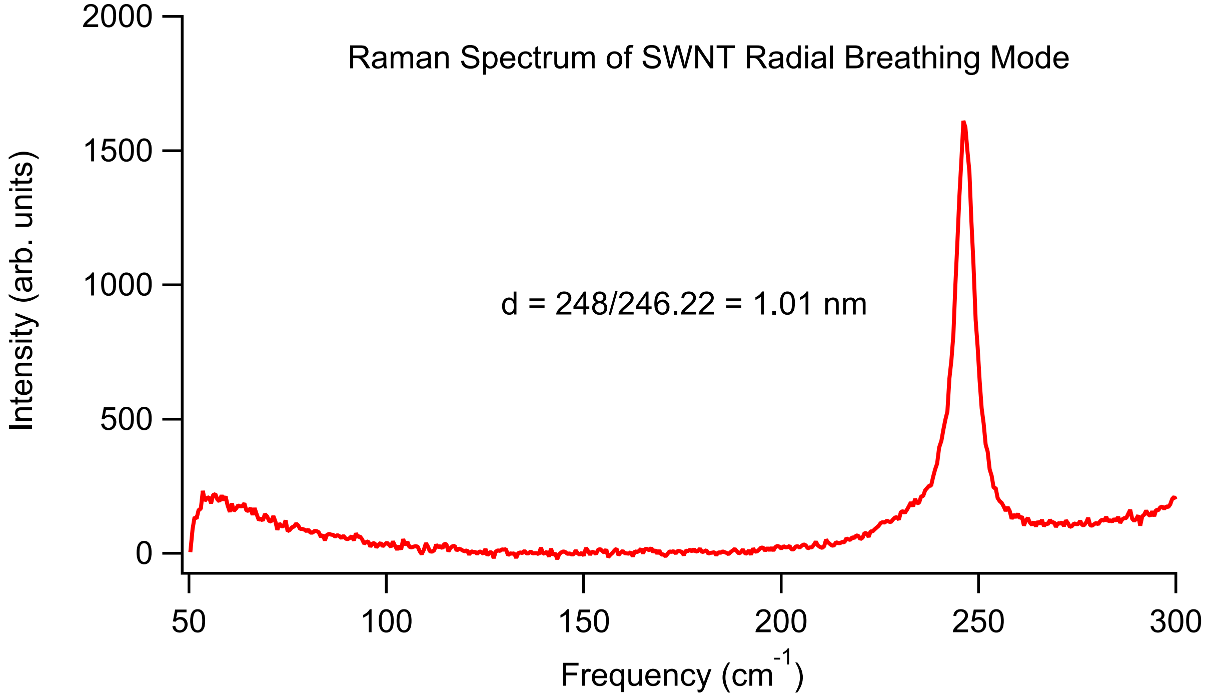


**Figure S2. Raman spectrum of the Radial Breathing Mode of the SWNT used in this research**

Figure S3 shows current data for Millipore water, no water, and a 1.0 M KCl solution in two reservoirs that were not connected by a SWNT. No pore-blocking events were observed for the time intervals shown nor for much longer time intervals. Several combinations of two reservoirs connected by a SWNT using Millipore water and several not connected by a SWNT using 1.0 M KCl were tested. In numerous current measurements using these reservoirs, no pore-blocking events were observed.

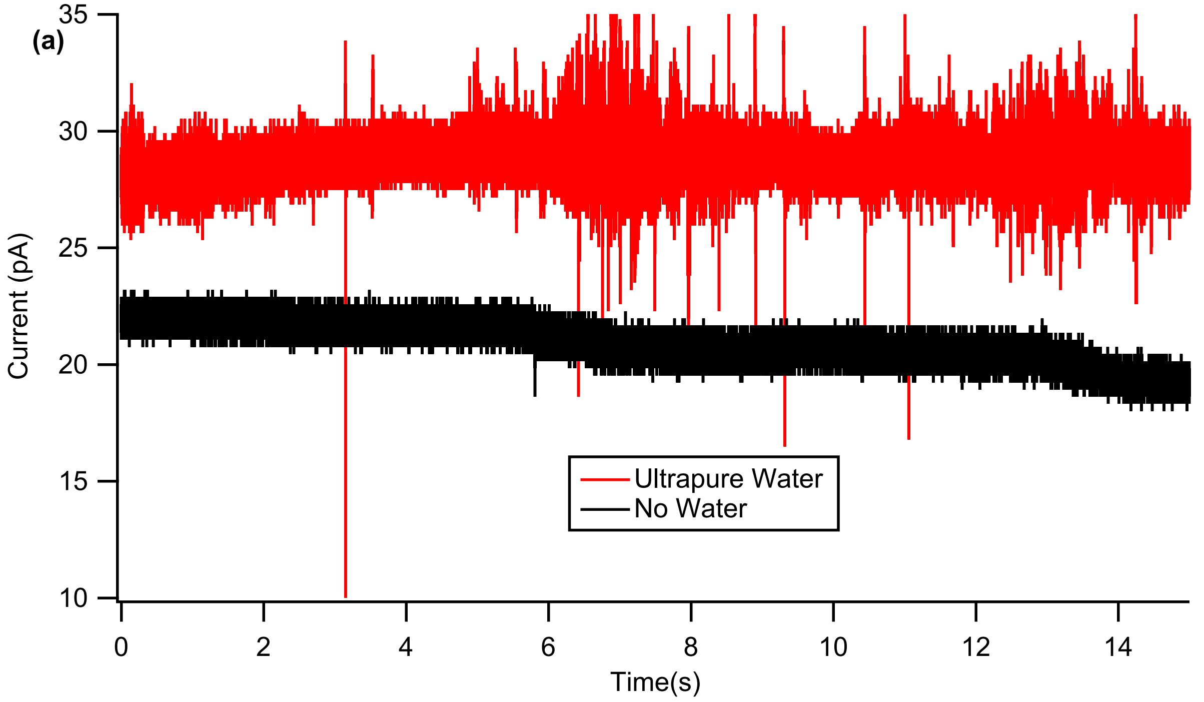
(a)
35
30
25
20
15
10
Current (pA)
0
2
4
6
8
10
12
14
Time(s)
Ultrapure Water
No Water

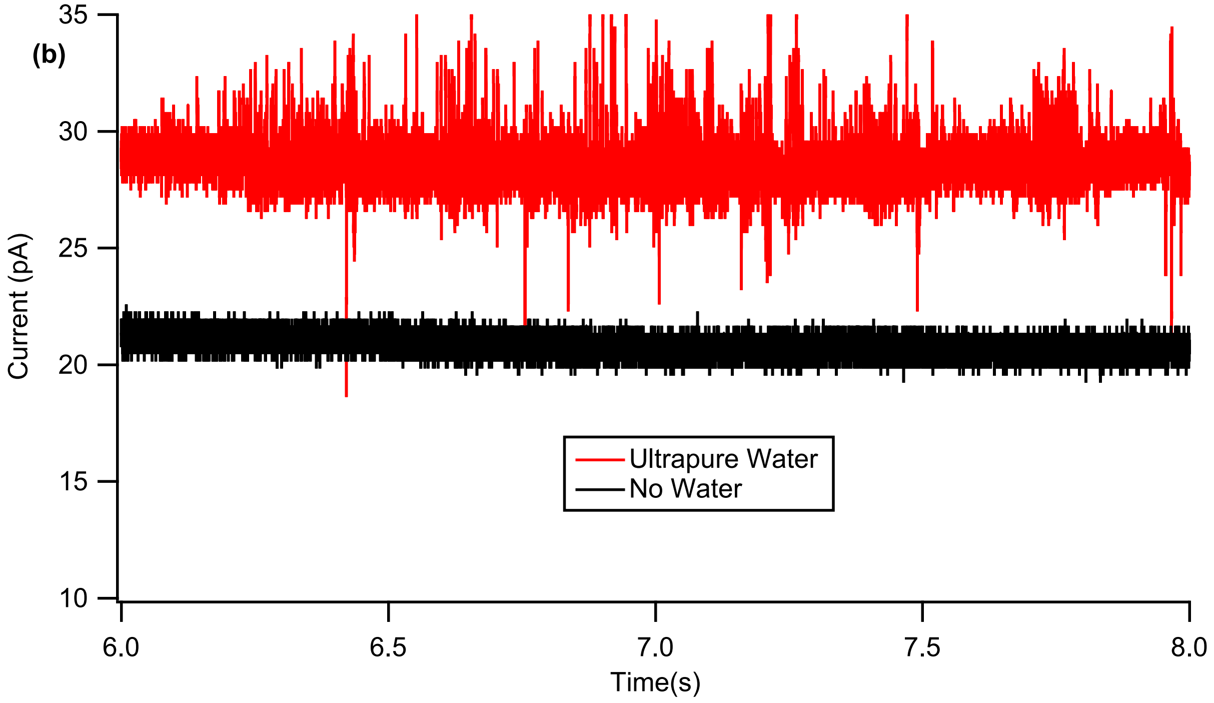
(b)
35
30
25
20
15
10
Current (pA)
6.0
6.5
7.0
7.5
8.0
Time(s)
Ultrapure Water
No Water

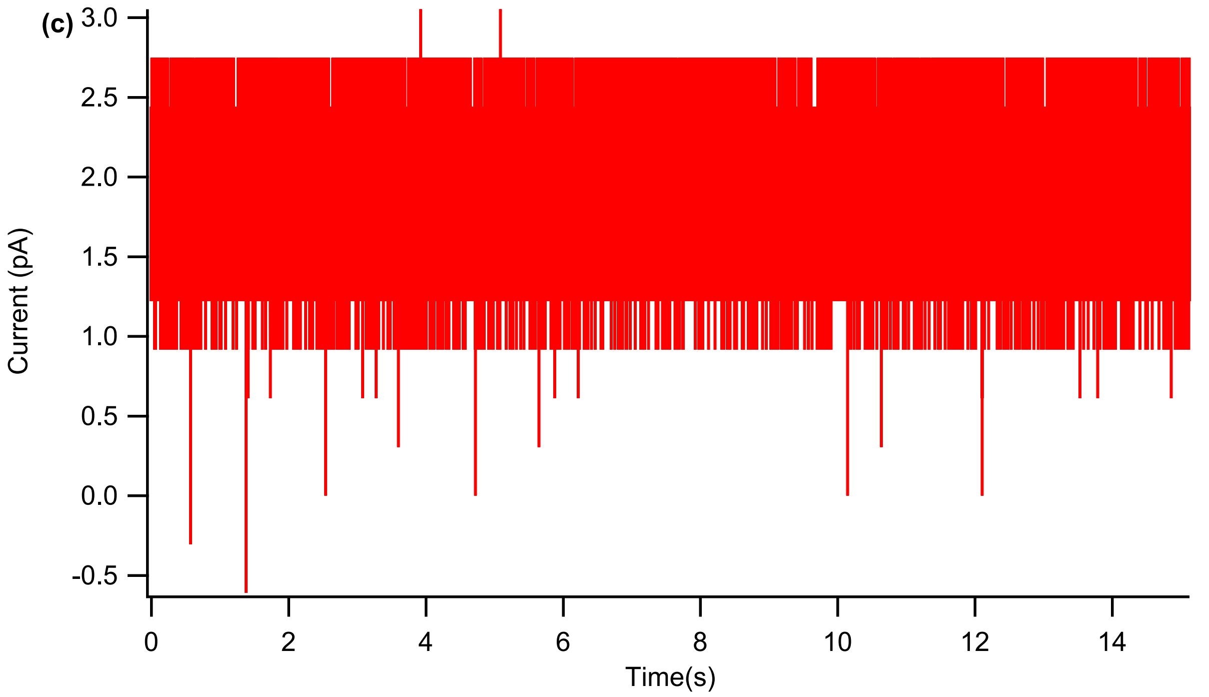


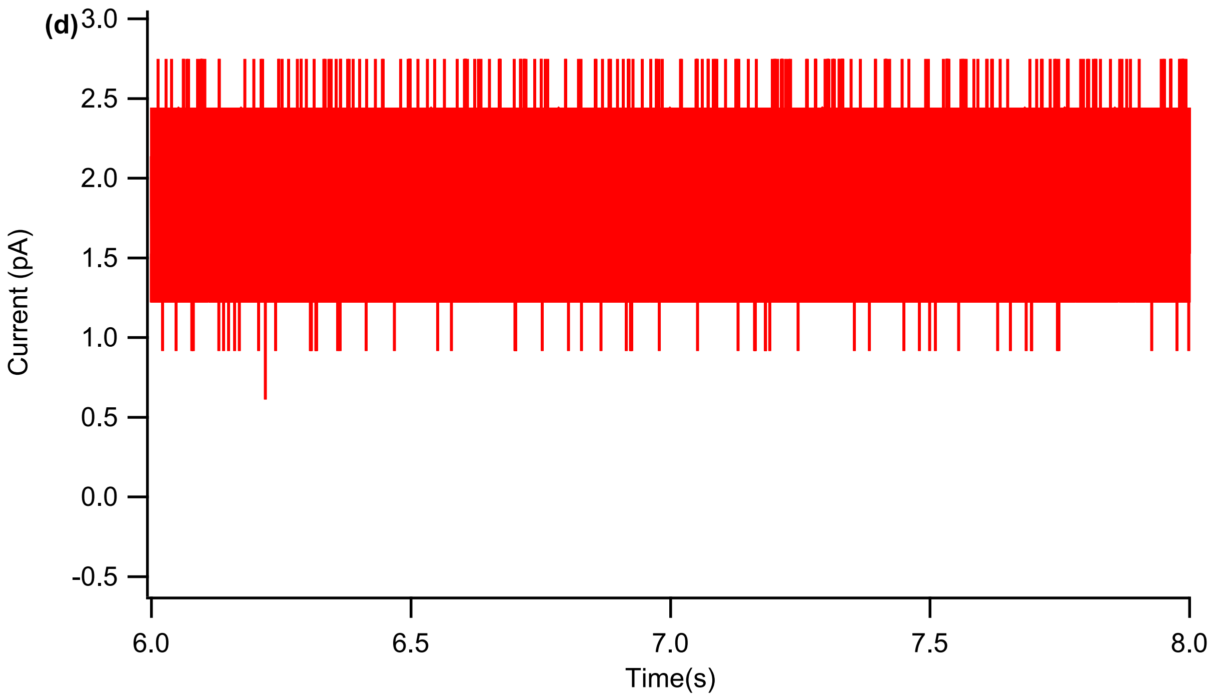


**Figure S3. Raw current data for (a) Millipore water and no water, 15-s interval, (b) Millipore water and no water, 2-s interval, (c) two wells not connected by a SWNT, 15-s interval, and (d) two wells not connected, 2-s interval. No pore-blocking events were observed.**

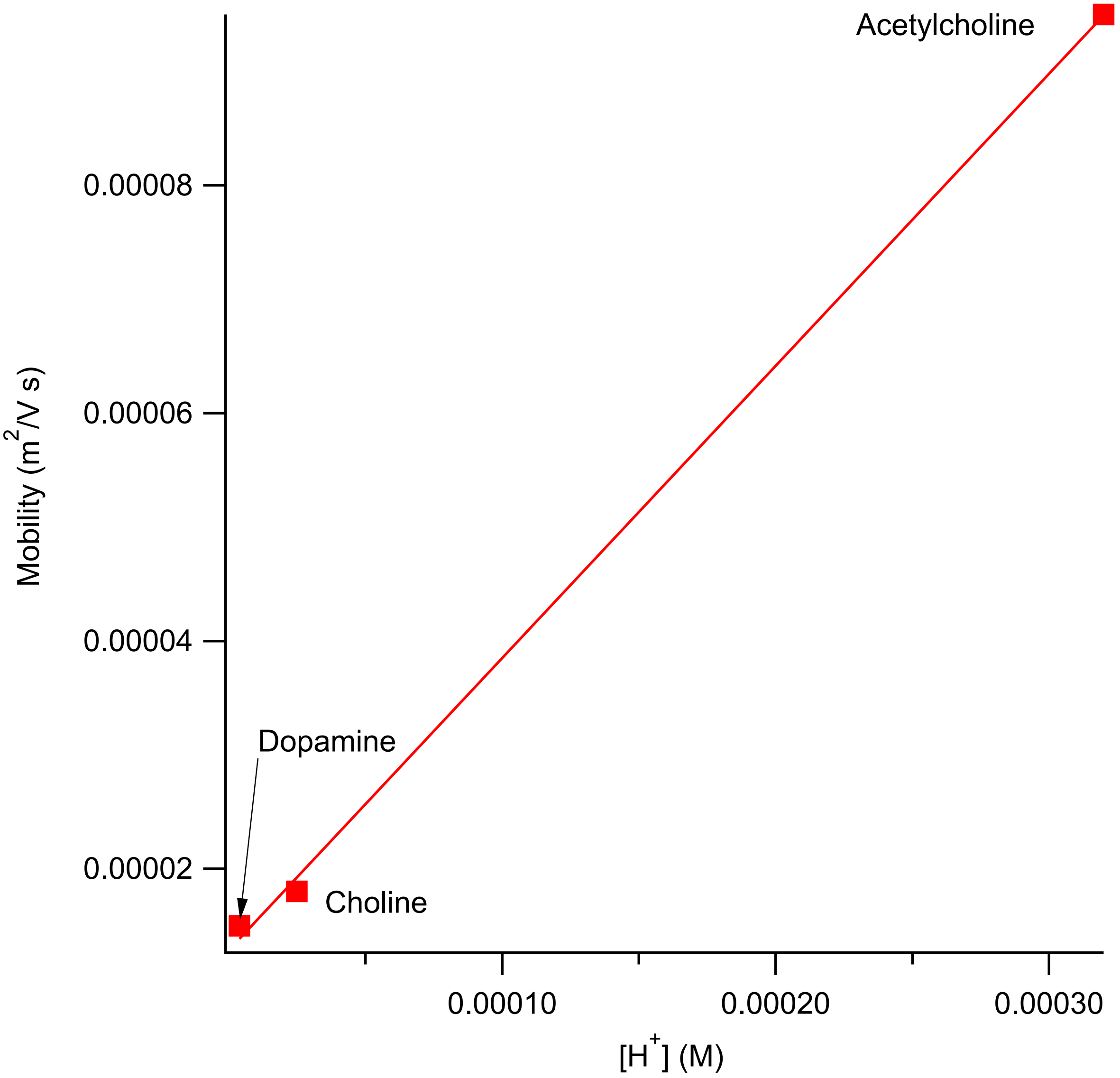


**Figure S4. Mobilities of 1.0 M solutions of dopamine, acetylcholine, and choline solutions as a function of the hydrogen ion concentration of those solutions.**

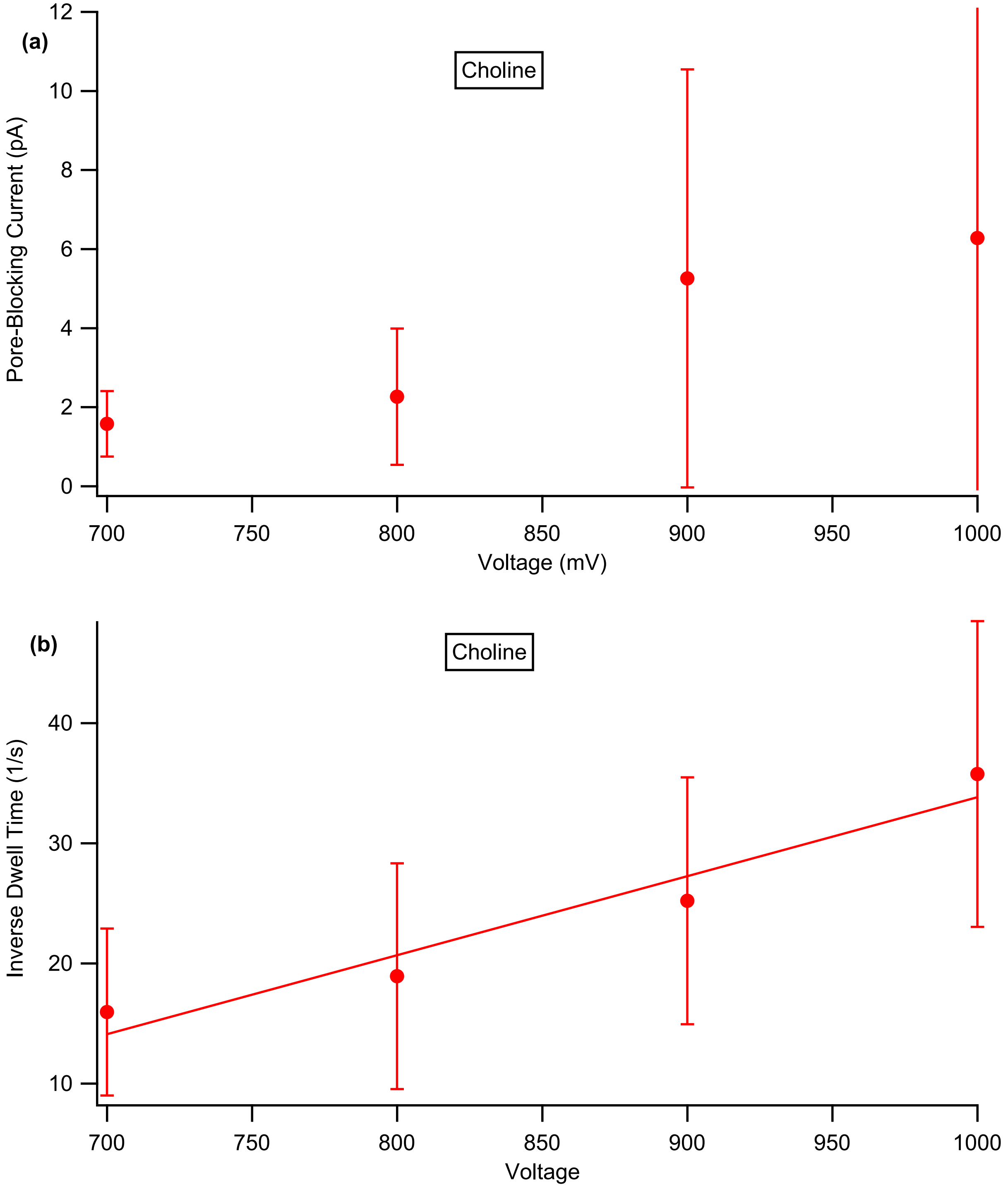


**Figure S5. For choline at pH = 4.6 (a) pore-blocking current, and (b) inverse dwell time as a function of voltage. The slope of the line is used to determine the mobility, as described in the text of the paper.**

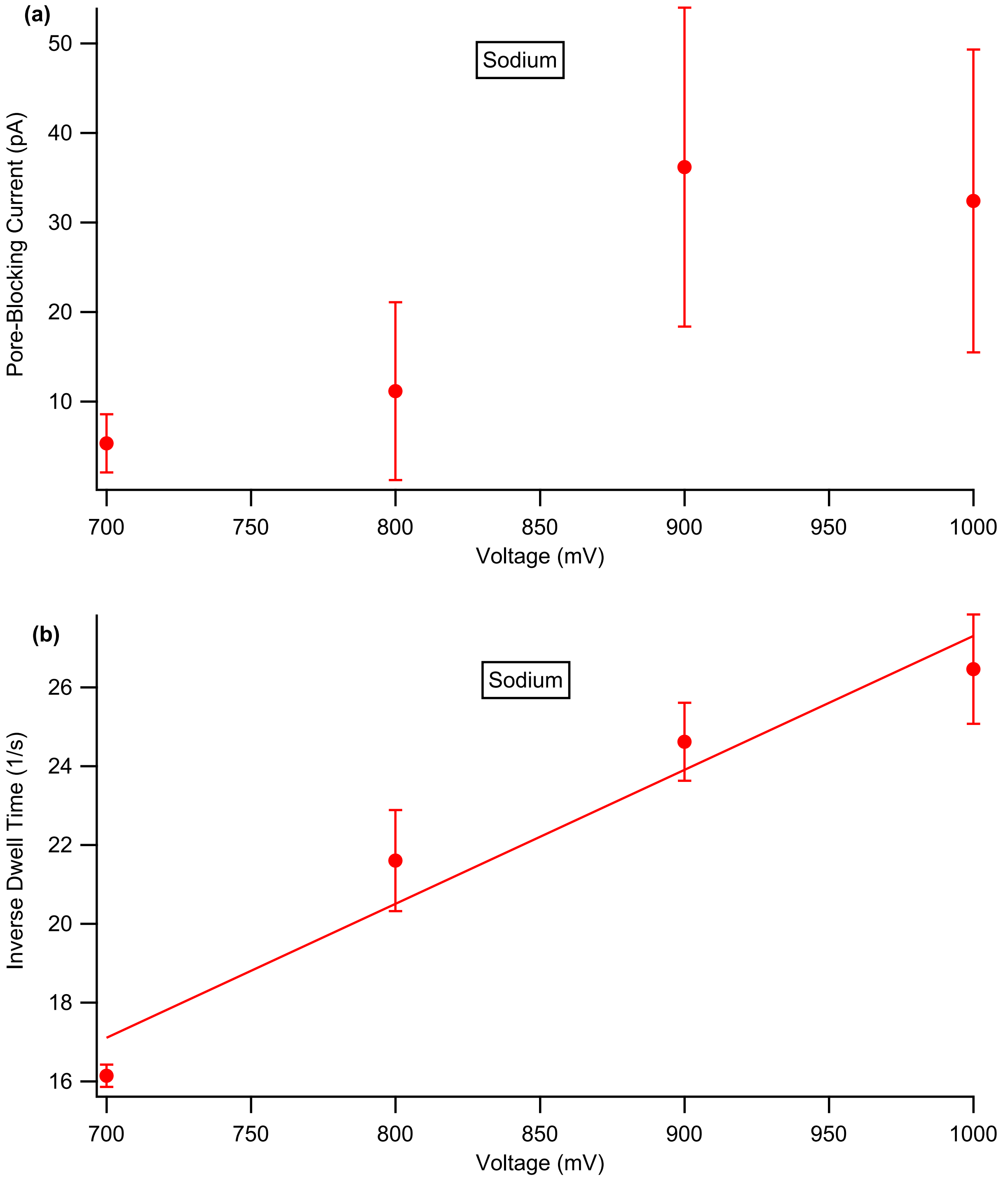


**Figure S6. For sodium at pH = 1.85 (a) pore-blocking current, and (b) inverse dwell time as a function of voltage. The slope of the line is used to determine the mobility, as described in the text of the paper.**

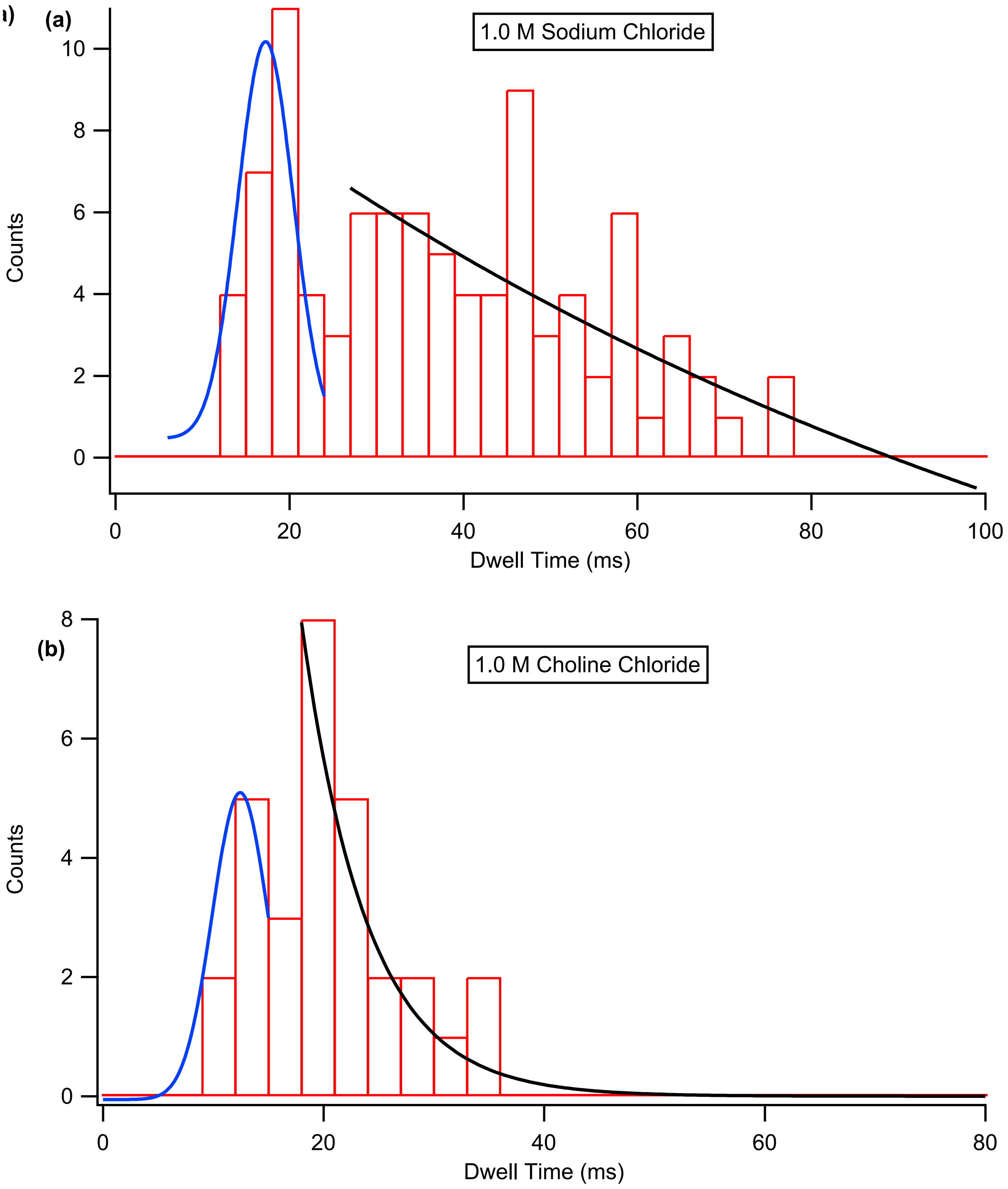
(a)
1.0 M Sodium Chloride
Counts
Dwell Time (ms)
(b)
1.0 M Choline Chloride
Counts
Dwell Time (ms)

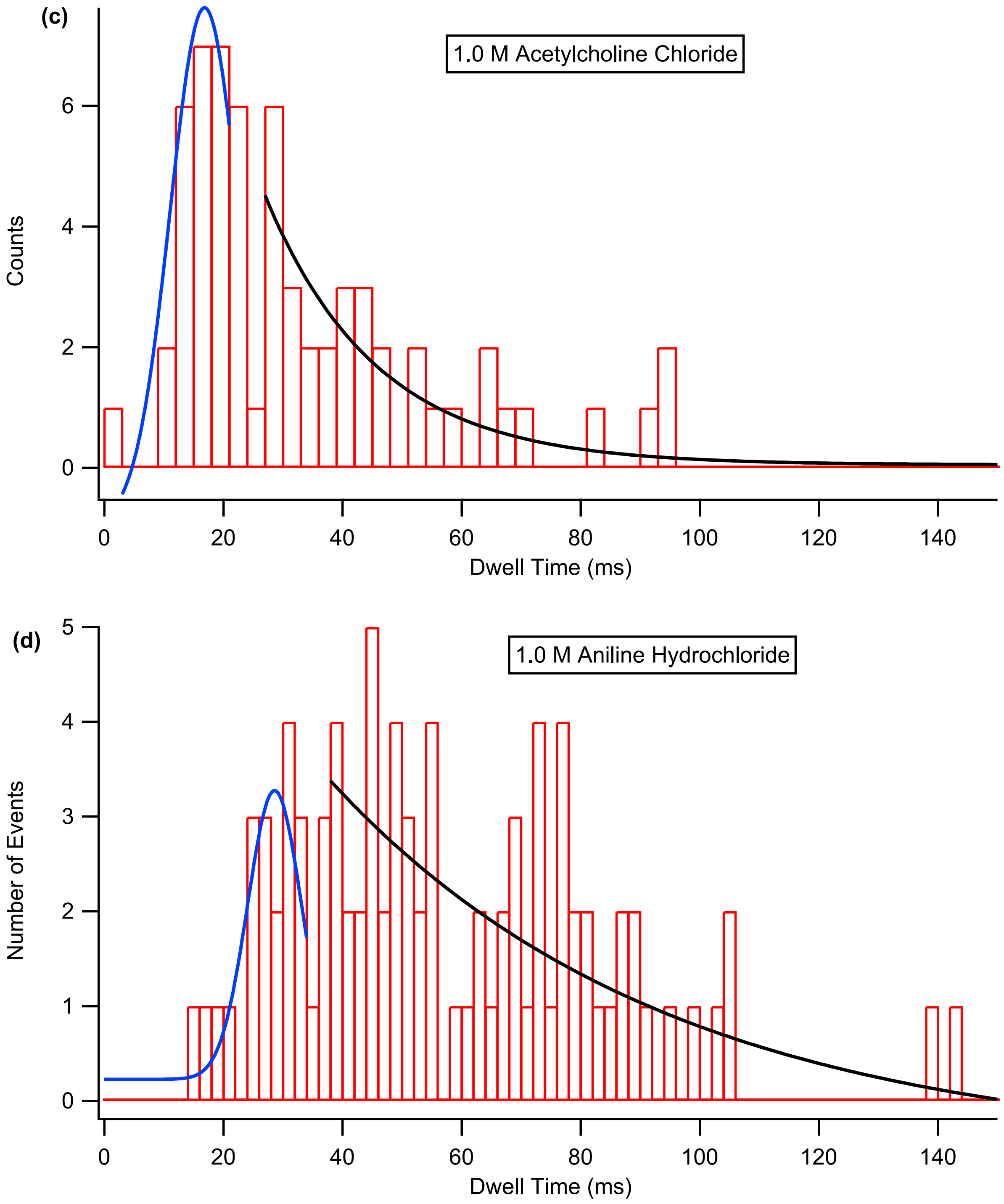


**Figure S7. Dwell time (ms) distributions for (a) 1.0 M sodium chloride at pH=1.85, (b) 1.0 M choline chloride at pH=1.30, (c) 1.0 M acetylcholine chloride at pH=1.21, and (d) 1.0 M aniline hydrochloride at pH=1.70. The short dwell times correspond to direct transport, and the blue line is a fit to guide the eye. The dark line is an exponential fit to the tail of the data, and the long dwell times correspond to molecule-ions that trapped and desorbed before exiting the SWNT. $k_{des}$ was calculated from the fit to the long dwell times.**

**Calculation of $k_{ads}$ and $k_{des}$**

For each amino acid, from the average mobility, the average velocity was calculated using

$$v = \mu E . \qquad (1)$$

Then, that average velocity and the average dwell time were used in the equation from the paper by Yeh and Hummer,[3]

$$\langle t \rangle = \frac{L}{v}\left(1 + \frac{k_{ads}}{k_{des}}\right) \qquad (2)$$

and the ratio $\frac{k_{ads}}{k_{des}}$ was determined. Using a fit to the tail of the dwell time distribution, $\tau \approx \frac{1}{k_{des}}$ was found, yielding $k_{des}$. Using the ratio, $k_{ads}$ was then calculated.

## References


1. Shuba, M. V.; Paddubskaya, A. G.; Kuzhir, P. P.; Maksimenko, S. A.; Ksenevich, V. K.; Niaura, G.; Seliuta, D.; Kasalynas, I.; Valusis, G., Soft Cutting of Single-Wall Carbon Nanotubes by Low Temperature Ultrasonication in a Mixture of Sulfuric and Nitric Acids. *Nanotechnology* **2012**, *23*, 495714.
2. Jorio, A.; Saito, R.; Hafner, J. H.; Lieber, C. M.; Hunter, M.; McClure, T.; Dresselhaus, G.; Dresselhaus, M. S., Structural (*N,M*) Determination of Isolated Single-Wall Carbon Nanotubes by Resonant Raman Scattering. *Phys. Rev. Lett.* **2001**, *86*, 1118-1121.
3. Yeh, I. C.; Hummer, G., Nucleic Acid Transport through Carbon Nanotube Membranes. *Proc Natl Acad Sci USA* **2004**, *101*, 12177-82.
